# Supplementary material for: Increased admission serum total bile acids can be associated with decreased 3-month mortality in patients with acute ischemic stroke
Source: Lipids Health Dis. 2022 Jan 22;21:15. doi: 10.1186/s12944-021-01620-8 (PMC8783998; doi:10.1186/s12944-021-01620-8)
Supplement: Supplementary file 1 — Additional file 1 [file 12944_2021_1620_MOESM1_ESM.pdf]

1176939511769395_TBA article （12.docx

WORD COUNT 3752
TIME SUBMITTED 12-DEC-2021 10:54AM
PAPER ID 80135040

Increased Admission Serum Total Bile Acids can be Associated with Decreased 3-month Mortality in Patients with Acute Ischemic Stroke

(TBA Associated with Mortality of AIS patients)

Abstract

Background: Bile acids (BAs) not only play an important role in lipid metabolism and atherosclerosis but also have antiapoptotic and neuroprotective effects. However, few studies have focused on the relationship of the total bile acid (TBA) levels with the severity and prognosis of acute ischemic stroke (AIS).

Objectives: The aim of this study was to investigate the potential associations of the fasting serum TBA levels on admission with the stroke severity, in-hospital complication incidence and 3 -month all-cause mortality in patients with AIS.

Methods: A total of 777 consecutive AIS patients were enrolled in this study and were divided into four groups according to the quartiles of the serum TBA levels on admission. Univariate and multivariate logistic regression analyses were used to explore the relationship between the fasting TBA levels and the stroke severity, in-hospital complications, and 3-month mortality in AIS patients.

Results: Patients in group Q3 had the lowest risk of severe AIS (NIHSS > 10) regardless of the adjustments for confounders ($P < 0.05$). During hospitalization, 115 patients (14.8%) had stroke progression (NIHSS score increased by ≥ 2), and 222 patients (28.6%) developed at least one complication, with no significant difference among the four groups ($P > 0.05$). There was no significant difference in the incidence of pneumonia, urinary tract infection (UTI), hemorrhagic transformation (HT),

gastrointestinal bleeding (GIB), seizures or renal insufficiency (RI) among the four groups ($P > 0.05$). A total of 114 patients (14.7%) died from various causes (including in-hospital deaths) at the 3-month follow-up, including 42 (21.3%), 26 (13.3%), 19 (9.9%) and 27 (13.9%) patients in groups Q1, Q2, Q3 and Q4 respectively, with significant differences ($P = 0.013$). After adjusting for confounding factors, the risk of death decreased (P -trend < 0.05) in groups Q2, Q3, and Q4 when compared with group Q1, and the OR values were 0.36 (0.16-0.80), 0.30 (0.13-0.70), and 0.29 (0.13-0.65), respectively.

Conclusions: TBA levels were inversely associated with the 3-month mortality of AIS patients but were not significantly associated with the severity of stroke or the incidence of complications.

Key words: Total bile acids; Acute ischemic stroke; Stroke severity; In-hospital complication; Mortality

Introduction

As the population ages, stroke has become the second leading cause of death(11.6% [10.8–12.2] of the total deaths in 2019) worldwide after ischemic heart disease [1] and is also associated with a high rate of disability and recurrence, which brings a great burden to society and families, especially in low- and middle-income countries [1, 2]. Ischemic stroke is the most prevailing type of stroke event. In 2019, acute ischemic stroke (AIS) was reported to account for 62.4% of all stroke events globally [1].

Primary intracerebral hemorrhage (PICH) accounted for approximately 27.9% of strokes, and subarachnoid hemorrhage (SAH) accounted for 9.7% of strokes [1]. Treatments such as early intravenous thrombolysis and endovascular treatment can allow the occluded blood vessels to be recanalized leading to blood reperfusion, which may reduce the infarct volume and effectively improve the overall prognosis of stroke patients. In addition, the therapeutic time window of reperfusion for AIS has been gradually extended owe to the development of neuroimaging techniques [3-9]. Unfortunately, the majority of AIS patients still fail to receive reperfusion treatment because they are outside of the time window, which affects the prognosis. Moreover, patients suffering from AIS, especially elderly and critically ill patients, commonly experience certain complications, such as poststroke pneumonia and gastrointestinal bleeding, which leads to a higher risk of early death, which is the result of a joint effect together with AIS [10-12].

Lipid metabolism disorders can cause cholesterol overload, leading to excessive deposition of lipid substances, such as low-density lipoprotein cholesterol (LDL-C), within the intima of the large and medium-sized arteries, which is considered the cause of the atherosclerosis incidence and the main risk factor for coronary heart disease, stroke, peripheral vascular disease, aortic aneurysm, and renal artery stenosis [13-17]. Studies have shown that excessive cholesterol in the human body can be converted into bile acids (BAs) and can be excreted from feces in the form of bile salts [13, 18, 19]. A large amount of bile acid excretion can prevent the development of atherosclerosis, while the reduction can lead to an increased risk of atherosclerosis and coronary heart

disease [20-22]. Researchers have also found that ursodeoxycholic acid (UDCA) facilitates the prevention of the occurrence of atherosclerosis and promotes plaque regression with dissolved cholesterol crystals [23]. Additionally, a 20-year prospective follow-up study showed that reduced bile acid excretion was an independent risk factor for stroke incidence and death [24].

In addition to being associated with lipid metabolism, bile acids were also reported to play a beneficial role in cellular protection and anti-apoptosis in rats with acute stroke and acute myocardial infarction [25-28], as well as in the reduction of glial cell activation in animal models of acute neuroinflammation [29]. A clinical trial found that there is a potential relationship between increased serum total bile acid (TBA) levels and a smaller hematoma volume during cerebral hemorrhage as well as a better outcome [30]. UDCA can be used to treat chronic heart failure by improving peripheral blood flow [31], while tauroursodeoxycholic acid (TUDCA) has antiapoptotic effects on a number of neurodegenerative diseases, including amyotrophic lateral sclerosis, Alzheimer's disease, Parkinson's disease and Huntington's disease [32].

To our knowledge, no study has evaluated serum TBA levels for associations with the clinical manifestations and early prognosis of patients with AIS. Here, we attempted to fill this gap by initially exploring the relationship between fasting TBA levels on admission and several AIS-related targets including stroke severity, in-hospital complications, and 3-month mortality.

Materials and Methods

Study population

A total of 777 consecutive AIS patients treated in the Department of Neurology, Zhangjiagang Hospital of Traditional Chinese Medicine (TCM) affiliated to Nanjing University of Chinese Medicine in China from April 2012 to January 2016 were eventually included in the study. The detailed inclusion and exclusion criteria are shown in **Table 1**.

The diagnosis of AIS was made by two or more neurologists after admission to our hospital based on the patient's medical history, clinical presentation, and brain computed tomography (CT) or magnetic resonance imaging (MRI) manifestations, according to World Health Organization (WHO) standards as follows: the development of a sudden focal or a complete neurological deficit, a neurological deficit lasting more than 24 h, exclusion of brain dysfunction caused by other nonvascular factors, and a diagnosis based on brain CT or MRI. All enrolled patients (n=983) had stable vital signs on admission without any severe disturbance of consciousness or any severe dysfunction of other organs. Patients who had more than 72 h from the onset to the admission (n=102) and those without TBA measurements within 24 h of admission (n=45) were excluded. In addition, patients who had severe hepatobiliary or renal disease prior to or on admission (n=17), underlying blood disease or cancer (n=16), any current infections or immune system disease (n=12) or who were lost to follow-up at 3 months of admission (n=14) were excluded as well (**Figure 1**).

Ethics statement

Approval of the Ethics Committee of Zhangjiagang TCM Hospital Affiliated to

Nanjing University of Chinese Medicine in China was obtained before starting the study (No. 2020-77-1), while the requirement for written informed consent was waived as this is a retrospective study and the data are anonymous. The study fully complied with the Declaration of Helsinki and obtained the required data from the clinical records without any clinical intervention for the protection of patient privacy.

Data collection

Baseline information was comprised of the demographic characteristics (such as sex, age) and known risk factors for cerebrovascular disease (such as stroke, hypertension, diabetes, atrial fibrillation, coronary heart disease, heart failure, smoking and drinking history). The time from onset to admission, stroke severity (National Institutes of Health Stroke Scale, NIHSS), previous thrombolytic therapy, clinical data and laboratory indexes on admission (such as systolic blood pressure, diastolic blood pressure, blood routine, serum TBA, liver function, blood glucose, blood lipids, creatinine, and uric acid) and in-hospital complications were recorded. The laboratory data were obtained in the emergency department before hospital admission or in the ward within 24 h after hospital admission. Blood routine data were obtained with XE-5000 (Mindray, Shenzhen, China). Biochemical data were obtained from fasting blood samples with Olympus AU5400 Automatic Analyzer (First Chemical Co., Ltd, Tokyo, Japan). All tests were completed by experts from the Laboratory Department of our hospital.

Outcome evaluation

The NIHSS score on admission was used to represent the severity of stroke on

admission. A NIHSS score greater than 10 was defined as a severe stroke, and a NIHSS score that had increased by more than 2 points was defined as stroke progression during hospitalization. Six complications of relatively high incidence, including pneumonia, urinary tract infection (UTI), hemorrhagic transformation (HT), gastrointestinal bleeding (GIB), seizures, and renal insufficiency, were included in the study. The definitions for these complications are described in **Table 2**. The three-month death rate was determined by telephone interviews of the patients or their families three months after the onset.

Statistical analysis

The quartiles of the TBA levels on admission were referenced to divide patients into four groups (Q1, ≤3.0 μmol/L; Q2, 3.0-5.7 μmol/L; Q3, 5.7-9.5 μmol/L; Q4, >9.5 μmol/L). SPSS software (Version 23.0; IBM, Armonk, NY, USA) was used for statistical analysis, and a two-tailed P value <0.05 was considered statistically significant.

Since four groups were generated with a total sample size ≥200 (each >100), continuous variables were analyzed in normality with the Kolmogorov-Smirnov test, and were represented by the mean (standard deviation) via one-way ANOVA in cases of all four groups are in normal distribution or the median (interquartile range) via Kruskal-Wallis test when one of the four groups did not conform to the normal distribution. Categorical variables were compared by the Chi-square test or Fisher's exact probability method.

The correlation analysis for the serum TBA with severe AIS on admission and the

3-month all-cause mortality was evaluated on univariate and multivariate logistic regression models. In the multivariate logistic regression model, the independence of TBA was identified after adjusting for covariates. The odds ratios (ORs) and 95% confidence intervals (CIs) were calculated for each group using the lowest quartile (Q1) of TBA as a reference. The potential confounders included age, sex, thrombolytic therapy, history of atrial fibrillation, the admission white blood cell (WBC) count and the platelet count for the TBA level and stroke severity. Age, sex, the NIHSS score on admission, progressive cerebral infarction and at least one complication during hospitalization, history of atrial fibrillation, and the admission WBC count are potential confounders for the TBA level and 3-month mortality.

Results

Baseline characteristics

In total, 777 eligible patients (420 males and 357 females) with AIS were enrolled in the study, and they had a mean age of 71 (62-78) years and a mean NIHSS score of 4 (3-8) on admission. The patients were assigned into groups Q1 (≤ 3.0 μmol/L, n = 197), Q2 (3.0-5.7 μmol/L, n = 195), Q3 (5.7-9.5 μmol/L, n = 191) and Q4 (> 9.5 μmol/L, n = 194) according to the quartiles of fasting serum TBA concentrations on admission, which were associated with NIHSS scores of 5, 5, 4 and 4, respectively, and there were no significant differences ($P = 0.389$) (**Table 3**). No significant differences were noted in the baseline demographic, clinical and laboratory parameters (including blood lipids) ($P > 0.05$), except for the history of atrial fibrillation (AF) and the admission white

blood cell (WBC) count ($P < 0.05$), among the four groups. Multiple comparisons showed that there was no significant difference in the AF rate between the Q1 and Q2 groups, while there was a significant difference between the other groups. A posthoc analysis found that the WBC count difference between the Q1 group and Q4 group was statistically significant ($P < 0.05$) and the WBC count in Q4 group was lower than in Q1 group (6.3 (5.1-7.9) vs 6.8 (5.6-8.5) $\times 10^9$/L).

Correlation between TBA and AIS severity

The numbers and proportions of severe AIS cases (NIHSS > 10) among the four groups were significantly different ($P = 0.029$), and they were much higher in group Q1 (n = 41, 20.8%) and group Q4 (n = 36, 18.6%), and were lower in group Q2 (n = 28, 14.4%) and group Q3 (n = 20, 10.5%) (**Table 4**). A binary logistic regression analysis showed that patients in group Q3 had a significantly lower risk of severe AIS than those in group Q1 (OR, 0.45; 95% CI, 0.25-0.79) before adjustments. In multivariate-adjusted models (Model 1 for age and sex, and Model 2 for age, sex, thrombolytic therapy, history of AF, WBC count, platelet count), compared to group Q1, patients in groups Q2 and Q3 had a lower risk of severe AIS, which was not reflected in group Q4. In addition, the p-trend was greater than 0.05 regardless of the adjustment for other confounding factors, and no significant trend was displayed.

Association between TBA and in-hospital complications

During hospitalization, 115 (14.8%) of the 777 patients had worsening of their AIS (NIHSS score increased by ≥ 2 points), but there was no significant difference among the four groups ($P = 0.584$). There were 222 (28.6%) patients that developed at least

one complication, with no significant difference among the groups ($P = 0.906$), and the incidence rates of pneumonia, UTI, HT, GIB, seizures, and renal insufficiency were 11.7%, 9.1%, 9.5%, 2.1%, 0.9%, and 2.4%, respectively, still with no significant difference among the four groups (all $P > 0.05$). The detailed results are shown in **Table 5**.

Correlation between TBA and 3-month all-cause mortality

The 3-month follow-up visits revealed that there were 114 deaths (14.7%) from various causes (including hospital deaths), and there were 42 (21.3%), 26 (13.3%), 19 (9.9%) and 27 (13.9%) deaths in groups Q1, Q2, Q3 and Q4, respectively, indicating significant differences ($P = 0.013$) (**Table 6**). In Model 2, with adjustments for sex, age, the NIHSS score on admission, AIS progression and the occurrence of at least one complication during hospitalization, the 3-month all-cause mortality decreased with the increase in serum TBA content. The OR values of groups Q2, Q3, and Q4 as compared to group Q1 were 0.36 (0.16-0.80), 0.35 (0.16-0.78), and 0.30 (0.14-0.66), respectively. In addition to the factors adjusted in Model 2, history of AF and the baseline WBC count were finally included in Model 3. In this case, the OR values of groups Q2, Q3, and Q4 were 0.36 (0.16-0.80), 0.30 (0.13-0.70), and 0.29 (0.13-0.65), respectively, compared to group Q1. In Model 2 and Model 3, both of the *P*-trend values were less than 0.05, indicating a decreased risk of 3-month mortality in reaction to the increase in serum TBA levels.

Discussion

In many animal experiments, bile acids, in addition to being a regulator of blood lipid and cholesterol content by participating in lipid metabolism, also act as signal molecules that activate different nuclear receptors, such as the farnesoid X receptor (FXR), pregnane X receptor (PXR), vitamin D receptor (VDR), and transmembrane G protein-coupled receptor 5 (TGR5), which reduce the risk of atherosclerosis via a variety of metabolic pathways in diverse tissues [15, 18, 33-35]. Bile acid chelates, such as coleswelen hydrochloride, can not only reduce the LDL-C levels, but also decrease the levels of hypersensitive C-reactive protein (hs-CRP) to prevent the development of atherosclerosis [36].

Bile acids also have anti-apoptosis and cellular protection effects. Andrew L. Rivard et al. [27] found reduced apoptosis and improved cardiac function in rats by TUDCA administration before myocardial infarction. In a rat model of acute stroke, bile acid TUDCA showed neuroprotective effects, and the underlying mechanism was proven with the involvement of enhanced cell apoptosis in response to inhibited mitochondrial disturbance and subsequent caspase activation [25]. In addition, TUDCA was found to negatively regulate Nrf2 signaling pathway to decrease lipid peroxidation, inflammation and apoptosis in acute cerebral infarction (ACI) rats [37]. TUDCA can not only reduce the cell apoptosis of rats with acute hemorrhagic stroke and protect the nerve from being damaged [26], but also reduce the activation of glial cells in animal models of acute neuroinflammation [29]. Joana D. Amaral et al. [28] reviewed the role of bile acids in the regulation process of apoptosis, which highlighted the anti-apoptotic effects of UDCA and TUDCA, as well as their potential application as new and

alternative drugs for the treatment of apoptosis-related diseases. All these certain evidences provide some basis for the conjecture that serum TBA may have a protective effect on AIS.

Comparisons with other studies and what does the current work add to the existing knowledge

An article published by Gideon Charach et al. in 2018 showed that diminished bile acid excretion is a risk factor for coronary artery disease [22]. At the same time, they also studied the in-hospital bile acid excretion of 68 men and 35 women admitted to the hospital between 1996 and 1998 for chest pain and suspected cardiac events and who were followed for up to 20 years [24]. They found a significantly higher average bile acid excretion in patients without stroke relative to those with stroke, while those with lower bile acid excretion had higher stroke incidence and mortality, suggesting that reduced bile acid excretion was also an independent risk factor for stroke incidence and death. A population-based cohort study in Taiwan demonstrated that cholecystectomy is related to a reduced risk of overall stroke, ischemic stroke, and hemorrhagic stroke [38]. Gallstones can cause bile excretion disorders and inflammation that is characterized by bile retention in the gallbladder. Lipid accumulation caused by decreased bile acid excretion, together with chronic inflammation, increases the risk of atherosclerosis, thereby increasing the risk of cerebral infarction [38]. Wenyuan Li et al. [34] analyzed the relationship between the fasting serum TBA levels and the occurrence and severity of coronary heart disease in a total of 7438 consecutive patients with suspected CAD, who had undergone coronary

angiography. They revealed that patients with CAD had lower fasting serum TBA levels than individuals without CAD. This indirectly established a link between the serum total bile acid levels and ischemic stroke, and this provides some support for the hypothesis that the serum total bile acid levels may play a protective role in ischemic stroke.

Most of the previous studies focused on the relationship between TBA and the occurrence and severity of coronary heart disease, cerebral infarction or other diseases. To our knowledge, this is the first clinical study to investigate the relationship between the admission serum TBA levels and stroke severity, in-hospital complication incidence, or short-term clinical outcomes in patients with AIS. In this study, the fasting serum TBA on admission showed a certain relationship with the 3-month clinical outcome，and low serum TBA levels were an independent risk factor for death within 3 months in patients with AIS. We speculated that the protective effect of TBA in this study may be related to cholesterol metabolism and its involvement as a signaling molecule in regulating various metabolic pathways in various tissues, as well as its neuroprotective and antiapoptotic effects. Bile acids can not only downregulate CYP7A1 expression by binding FXR but can also restrict the continuous synthesis of bile acids and maintain the homeostasis of bile acids through a feedback mechanism[39]. Moreover, FXR and TGR5 can regulate glucose and lipid metabolism, activate the AKT pathway to stimulate glycogen synthesis and inhibit gluconeogenesis to mimic insulin regulation of glucose metabolism[18]. In addition, TGR5 activation can also reduce chronic inflammation, improve insulin resistance and inhibit atherosclerosis by inhibiting

systemic inflammation and macrophage infiltration in adipose tissue [18, 40].

We also found that patients in the Q4 group with the highest bile acid had lower WBC counts than those in the Q1 group, indicating that reduced inflammation may play a role in the protective effects of bile acids. We can further clarify the relevant mechanisms through animal experiments and the measurement of biomarkers in patients, such as interleukin, hs-CRP and other inflammatory indicators. At the same time, we can continue to carry out longer term follow-up studies, including the evaluation of patient survival, functional prognosis, recurrence of stroke and occurrence of cardiovascular events, for further research.

Study strengths and limitations

This study has the following strengths：(1) This study is the first to identify an association between high fasting serum TBA levels on admission and reduced mortality within three months after stroke in patients with AIS. (2) Although the serum TBA levels were not significantly correlated with the blood lipid levels (including triglycerides, total cholesterol, low-density lipoprotein cholesterol, and high-density lipoprotein cholesterol), the stroke severity, or in-hospital complication incidence, they were correlated with the incidence of AF and the WBC count, which may be a direction for research in future studies.

However, there are still some limitations: (1) This is only a single-center retrospective study with a small sample size limited to Chinese patients, and some results may vary among different populations. Though studies have shown that the characteristics and prevalence of cerebrovascular and cardiovascular risk factors in the

Asian population are similar to those of other large contemporary trials and real-world registries that also include other ethnicities [41-43], further studies involving different populations and more centers are needed to support our findings. (2) Although our model was adjusted for several covariates that might have an impact on the outcomes, there are still some possible influencing factors that have not been collected. (3) This study did not follow up on the functional outcomes in patients with AIS who survived more than 3 months; thus, this study was unable to determine the effect of serum TBA on the functional recovery. (4) As long-term follow-up has not yet been completed, the long-term effects of serum TBA cannot be determined in this study. (5) In this study, we only measured the serum total fasting TBA, without specific components of bile acids.

Conclusion

This study shows that the admission fasting serum TBA levels were inversely associated with the 3-month mortality of AIS patients but were not significantly associated with the severity of stroke or the incidence of complications. This suggests that serum TBA levels may be a simple, cost-effective and readily available biomarker with additional predictive value for the prognosis of patients with AIS. Bile acids measurement at admission can help clinicians predict the prognosis of AIS patients, supplementation with bile acids during hospitalization, such as UDCA, may be beneficial to the prognosis of AIS patients.

1176939511769395_TBA article（12.docx

ORIGINALITY REPORT

64%
SIMILARITY INDEX

PRIMARY SOURCES

1	Lingling Huang, Ge Xu, Rong Zhang, Jiahui Ji, Yadong Wang, Yaming Sun. "Increased Admission Serum Total Bile Acids can be Associated with Decreased 3-month Mortality in Patients with Acute Ischemic Stroke", Research Square Platform LLC, 2021<br>Crossref Posted Content	2636 words — 62%
2	Wenyuan Li, Shan Shu, Lele Cheng, Xiang Hao, Lijun Wang, Yue Wu, Zuyi Yuan, Juan Zhou. "Fasting serum total bile acid level is associated with coronary artery disease, myocardial infarction and severity of coronary lesions", Atherosclerosis, 2020<br>Crossref	61 words — 1%
3	Wencai Jiang, Meixiang Chen, Jianyu Huang, Yu Shang et al. "Proteinuria is independently associated with carotid atherosclerosis: a multicentric study", BMC Cardiovascular Disorders, 2021<br>Crossref	19 words — < 1%
4	nutrition.moh.gov.my<br>Internet	13 words — < 1%

EXCLUDE QUOTES	ON	EXCLUDE MATCHES	OFF
EXCLUDE BIBLIOGRAPHY	ON		
