# Supplementary material for: Increased admission serum total bile acids can be associated with decreased 3-month mortality in patients with acute ischemic stroke
Source: Lipids Health Dis. 2022 Jan 22;21:15. doi: 10.1186/s12944-021-01620-8 (PMC8783998; doi:10.1186/s12944-021-01620-8)
Supplement: Supplementary file 2 — Additional file 2 [file 12944_2021_1620_MOESM2_ESM.pdf]

Editing Certificate

This document certifies that the manuscript

Increased Admission Serum Total Bile Acids can be Associated with Decreased 3-month Mortality in Patients with Acute Ischemic Stroke

prepared by the authors

Lingling Huang, Ge Xu, Rong Zhang, Yadong Wang, Jiahui Ji, Fengdan Long, Yaming Sun

was edited for proper English language, grammar, punctuation, spelling, and overall style by one or more of the highly qualified native English speaking editors at AJE.

This certificate was issued on **December 9, 2021** and may be verified on the AJE website using the verification code **D76D-7155-79A0-6F2E-304P** .

Neither the research content nor the authors' intentions were altered in any way during the editing process. Documents receiving this certification should be English-ready for publication; however, the author has the ability to accept or reject our suggestions and changes. To verify the final AJE edited version, please visit our verification page at aje.com/certificate. If you have any questions or concerns about this edited document, please contact AJE at support@aje.com.

AJE provides a range of editing, translation, and manuscript services for researchers and publishers around the world. For more information about our company, services, and partner discounts, please visit aje.com.
