# Supplementary material for: Increased admission serum total bile acids can be associated with decreased 3-month mortality in patients with acute ischemic stroke
Source: Lipids Health Dis. 2022 Jan 22;21:15. doi: 10.1186/s12944-021-01620-8 (PMC8783998; doi:10.1186/s12944-021-01620-8)
Supplement: Supplementary file 4 — Additional file 4 [file 12944_2021_1620_MOESM4_ESM.pdf]

SPRINGER NATURE

Change of authorship request form - Journals (pre-acceptance)

Important information. Please read.

- This form should be used by authors to request any change in authorship (adding/deleting authors) including changes in corresponding authors. This form should not be used for name changes. Please fully complete all sections. Use black ink and block capitals and provide each author's full name with the given name first followed by the family name.
- By signing this declaration, all authors guarantee that the order of the authors are in accordance with their scientific contribution, if applicable as different conventions apply per discipline, and that only authors have been added who made a meaningful contribution to the work.
- Please note, in author collaborations where there is formal agreement for representing the collaboration, it is sufficient for the representative or legal guarantor (usually the corresponding author) to complete and sign the Authorship Change Form on behalf of all authors, **next to the added/removed author(s). (Complete Section 3, followed by Section 6.)**
  In author collaborations where there is no formal agreement for representing the collaboration and **there are more than 10 authors**, one may sign for all, provided the signer appends correspondence that attests that each of the authors have agreed to the change **and the added/removed authors sign the form. (Complete Section 3, followed by Section 6.)**
- Please note, we cannot investigate or mediate any authorship disputes. If you are unable to obtain agreement from all authors (including those who you wish to be removed) you must refer the matter to your institution(s) for investigation. Please inform us if you need to do this.
- If you are not able to return a fully completed form within **30 days** of the date that it was sent to the author requesting the change, we may have to withdraw your manuscript. We cannot publish manuscripts where authorship has not been agreed by all authors (including those who have been removed).
- Incomplete forms will be rejected.
- Please return/upload this form, fully completed, to the Journals Editorial Office. The Journal and/or Publisher will consider the information you have provided to decide whether to approve the proposed change in authorship. We may decide to contact your institution for more information or undertake a further investigation, if appropriate, before making a final decision.

SPRINGER NATURE

Change of authorship request form - Journals (pre-acceptance)

Section 1: Please provide the current title of manuscript

Manuscript ID no.: LHAD-D-21-00584R1

Title: Increased Admission Serum Total Bile Acids can be Associated with Decreased 3-month Mortality in Patients with Acute Ischemic Stroke

Section 2: Please provide the previous authorship, in the order shown on the manuscript before the changes were introduced. Please indicate the corresponding author by adding (CA) behind the name.

	First name(s)	Family name	ORCID or SCOPUS id, if available
1st author	Lingling	Huang	
2nd author	Ge	Xu	
3rd author	Rong	Zhang	
4th author	Yadong	Wang	
5th author	Jiahui	Ji	
6th author	Yaming (CA)	Sun (CA)	

Please use an additional sheet if there are more than 10 authors.

SPRINGER NATURE Springer Nature is one of the world's leading global research, educational and professional publishers, created in May 2015 through the combination of Nature Publishing Group, Palgrave Macmillan, Macmillan Education and Springer Science+Business Media.

SPRINGER NATURE

Change of authorship request form - Journals (pre-acceptance)

Section 3: Please provide a justification for change. Please use this section to explain your reasons for changing the authorship of your manuscript, e.g. what necessitated the change in authorship? Please refer to the (journal) policy pages for more information about authorship. Please explain why omitted authors were not originally included and/or why authors were removed on the submitted manuscript.

This newly added author was originally in the list of authors when we first submitted the manuscript. Our manuscript stated that Fengdan Long (FL) was the sixth author and her contribution, but due to negligence in the submission, she was not added or was deleted by mistake when we use the submission system clicked to add author information, and we did not check this at the time.

Section 4: Proposed new authorship. Please provide your new authorship list in the order you would like it to appear on the manuscript. Please indicate the corresponding author by adding (CA) behind the name. <u>If the Corresponding Author has changed, please indicate the reason under section 3</u>.

	First name(s)	Family name (this name will appear in full on the final publication and will be searchable in various abstract and indexing databases)	Affiliated institute	E-mail address
1st author	Lingling	Huang	Zhangjiagang TCM Hospital Affiliated to Nanjing University of Chinese Medicine in China	54hll00@163.com
2nd author	Ge	Xu	Zhangjiagang TCM Hospital Affiliated to Nanjing University of Chinese Medicine in China	georgia-xu@hotmail.com
3rd author	Rong	Zhang	Zhangjiagang TCM Hospital Affiliated to Nanjing University of Chinese Medicine in China	zhronghappy@163.com
4th author	Yadong	Wang	Zhangjiagang TCM Hospital Affiliated to Nanjing University of Chinese Medicine in China	yadong2006@126.com
5th author	Jiahui	Ji	Zhangjiagang TCM Hospital Affiliated to Nanjing University of Chinese Medicine in China	jijiahui0728@126.com
6th author	Fengdan	Long	Zhangjiagang TCM Hospital Affiliated to Nanjing University of Chinese Medicine in China	adan02@126.com
7th author	Yaming (CA)	Sun (CA)	Zhangjiagang TCM Hospital Affiliated to Nanjing University of Chinese Medicine in China	sunyaming2001@126.com

Please use an additional sheet if there are more than 10 authors.

SPRINGER NATURE Springer Nature is one of the world's leading global research, educational and professional publishers, created in May 2015 through the combination of Nature Publishing Group, Palgrave Macmillan, Macmillan Education and Springer Science+Business Media.

SPRINGER NATURE

Change of authorship request form - Journals (pre-acceptance)

Section 5: Author contribution, Acknowledgement and Disclosures. Please use this section to provide a new disclosure statement and, if appropriate, acknowledge any contributors who have been removed as authors and ensure you state what contribution any new authors made (if applicable per the journal or book (series) policy). Please ensure these are updated in your manuscript - after approval of the change(s) - as our production department will not transfer the information in this form to your manuscript.

New acknowledgements:
N/A

New Disclosures (financial and non-financial interests, funding):
This work was supported by Special research of Suzhou industrial technology Innovation (SYSD2017006).

New Author Contributions statement (if applicable per the journal policy):
RZ and YS provided funding and designed the study. GX, FL and YW collected the data. LH, FL and JJ were involved in data cleaning, follow-up and verification. YS revised the article.
All authors have read and approved the final manuscript.

State 'Not applicable' if there are no new authors.

SPRINGER NATURE # Change of authorship request form - Journals (pre-acceptance)

Section 6: Declaration of agreement. *All* authors, unchanged, new and removed *must* sign this declaration.
(NB: Please print the form, (docu)-sign and return/upload a scanned copy. Please note that signatures that have been inserted as an image file are acceptable as long as it is handwritten. Typed names in the signature box are unacceptable.) *** Please delete as appropriate. Delete all of the bold if you were on the original authorship list and are remaining as an author.**

	First name	Family name		Signature	Date
1st author	Lingling	Huang	I agree to the proposed new authorship shown in section 4 and the proposed change in corresponding author	Ling Ling Huang	2021.12.13
2nd author	Ge	Xu	I agree to the proposed new authorship shown in section 4 and the proposed change in corresponding author	Ge Xu	2021.12.13
3rd author	Rong	Zhang	I agree to the proposed new authorship shown in section 4 and the proposed change in corresponding author	Rong Zhang	2021.12.13
4th authors	Yadong	Wang	I agree to the proposed new authorship shown in section 4 and the proposed change in corresponding author	Yadong Wang	2021.12.13
5th author	Jiahui	Ji	I agree to the proposed new authorship shown in section 4 and the proposed change in corresponding author	Jiahui Ji	2021.12.13
6th author	Fengdan	Long	I agree to the proposed new authorship shown in section 4 **and the addition of my name to the authorship list** and the proposed change in corresponding author	Fengdan Long	2021.12.13
7th author	Yaming (CA)	Sun (CA)	I agree to the proposed new authorship shown in section 4 and the proposed change in corresponding author	Yaming Sun	2021.12.13

SPRINGER NATURE Springer Nature is one of the world's leading global research, educational and professional publishers, created in May 2015 through the combination of Nature Publishing Group, Palgrave Macmillan, Macmillan Education and Springer Science+Business Media.

SPRINGER NATURE **Change of authorship request form - Journals** (pre-acceptance)

In case of author collaborations with formal agreement:

	Name of consortium/consortia	First name	Family name		Signature	Date
Representative/legal guarantor				I agree to the proposed new authorship shown in section 4 **/and the addition/removal*of my name to the authorship list** /and the proposed change in corresponding author		

Both added/removed authors should complete the information in the first table under Section 6.

---- End of form ----

SPRINGER NATURE Springer Nature is one of the world's leading global research, educational and professional publishers, created in May 2015 through the combination of Nature Publishing Group, Palgrave Macmillan, Macmillan Education and Springer Science+Business Media.
