# Supplementary material for: Increased admission serum total bile acids can be associated with decreased 3-month mortality in patients with acute ischemic stroke
Source: Lipids Health Dis. 2022 Jan 22;21:15. doi: 10.1186/s12944-021-01620-8 (PMC8783998; doi:10.1186/s12944-021-01620-8)
Supplement: Supplementary file 3 — Additional file 3 [file 12944_2021_1620_MOESM3_ESM.docx]

Ms. Ref. No.: LHAD-D-21-00584

Title: **Increased Admission Serum Total Bile Acids can be Associated with Decreased 3-month Mortality in Patients with Acute Ischemic Stroke**

**Lipids in Health and Disease**

Dear Prof. A. M. Abd El-Aty:

Thank you very much for your letter and comments concerning our manuscript. All those comments are valuable and very helpful for improving our paper. We have endeavored to revise the manuscript according to the editor’s and reviewers’ suggestions and would like to re-submit it for your consideration. The amendments are marked in yellow in the revised manuscript and point by point responses to the editor’s and reviewers’ comments are outlined in this letter.

We would like to express our great appreciation to the editor and reviewers for comments on our paper. We hope that the revised manuscript has adequately addressed the editor’s and reviewers’ concerns and would be suitable for publication. We look forward to hearing from you regarding the suitability of the manuscript for publication in ***Lipids in Health and Disease***.

Thank you and best regards.

Yaming Sun, M.D., Ph.D.

Department of Neurology

Zhangjiagang TCM Hospital Affiliated to Nanjing University of Chinese Medicine in China, Suzhou 215600, China

Phone: (86)-13773241027

Fax: (86) 0512-5638-0372

E-mail: sunyaming2001@126.com

**Editor:**

1. The overall similarity index of the MS should be < 10%. Check this through running the MS on iThenticate (neither Turnitin nor copykiller, etc.). Attach the proof report as Suppl. Materials.

**Response:** Through running the article on iThenticate, the overall similarity index of the article is < 10%. (Although the repetition rate check showed that the overall similarity rate was 64%, this 62% similar article was actually uploaded to the preprint when we first submitted the manuscript.)

2. After incorporating all the amendments, the MS should be checked by a native speaker for grammar and syntax errors. A certificate of language editing should be uploaded as Suppl. materials.

**Response:** After incorporating all the amendments, the article was checked by a native speaker (AJE website) for grammar and syntax errors. The certificate of language editing has been uploaded as Suppl. materials.

3. All amendments, including language editing, should be highlighted “yellow”. State clearly line numbering when responding to each query.

**Response:** All amendments, including language editing, were highlighted “yellow”. and we stated clearly line numbering when responding to each query.

4. Make sure that the reference lists were prepared according to journal format if any References in the text should typically run with the text. i.e. not superscript.

**Response:** We make sure that the reference lists were prepared according to journal format and the References in the text are run with the text.

5. Abbreviations MUST be stated in full acronym before being abbreviated throughout the MS.

**Response:** Abbreviations have been stated in full acronym before being abbreviated throughout the article.

6. P letter for statistical analysis should be uppercase - italic face letter. Revise throughout the MS.

**Response:** All the P letter for statistical analysis is uppercase - italic face letter.

7. Don’t use “we, our”. Use impersonal phrasing throughout the text.

**Response:** We have revised the "we, our" to non-personal wording such as "this study, it" and so on.

8. Check spaces and punctuation throughout the MS.

**Response:** We have checked the spaces and punctuation throughout the article.

9. Line 72: In 2017, acute ischemic stroke (AIS) was reported ...., update to the curret.

**Response:** Thank you for this comment. We have updated the references to the most recent article published in 2021 in *Lancet Neurol*. See line 68-75 for details in the article.

10. Add clearance No for Ethical approval.

**Response:** The clearance No for Ethical approval is No. 2020-77-1. Line 145 in the article.

11. Full vendor details should include company, city (state), country. Revise throughout the MS.

**Response:** We add the company, city (state), country in vendor details. Line 160-162 in the article.

12. Study strengths and limitations should be in a separate section, headed as addressed. It should be ahead of the conclusion. Start with the strength of the study, followed by limitations.

**Response:** We add the Study strengths and limitations as a separate section of Discussion. See line 362-385 for details in the article.

13. A section headed as “Comparisons with other studies and what does the current work add to the existing knowledge” should be added before “Study strengths and limitations. The novel aspect of the study should be justified. Make sure not to repeat the same information stated elsewhere throughout the text.

**Response:** A section headed as “Comparisons with other studies and what does the current work add to the existing knowledge” has been added before “Study strengths and limitations. See line 312-361 for details in the article.

14. What are the clinical relevance and future perspective? Add this to the conclusion section. The clinical relevance cannot be summarized in "more prospective studies are needed...", that has no clinical relevance. Would you please point at how your findings impact the care of patients?

**Response:** In the Discussion (line 392-395), we add the clinical relevance and future perspective “Bile acids measurement at admission can help clinicians predict the prognosis of AIS patients, supplementation with bile acids during hospitalization, such as UDCA, may be beneficial to the prognosis of AIS patients”.

15. Make sure that Tables/Figs/references were cited in order without missing any.

**Response:** We make sure that the Tables/Figs/references corresponds to the content in the article.

16. Make sure that Table legends and Fig. captions are provided without missing any.

**Response:** We make sure that the Table legends and Fig. captions are provided without missing any.

17. Upload 2 files; edited with all amendments highlighted “yellow” and clean version.

**Response:** We make sure to upload 2 files: edited with all amendments highlighted “yellow” and clean version.

**Reviewer #1:**

**General comments:**

1. The paper is not particularly well written. The text is sometimes challenging to understand. The manuscript presentation should be improved (including the abstract). Please, correct typos and grammatical errors.

**Response:** Thank you for these constructive comments. The manuscript presentation has been improved (including the abstract) by a native speaker (AJE website) for typos and grammatical errors.

2. While the paper is within the word limit, I feel that readability would be enhanced by being more succinct and shortening the word count.

**Response:** Thank you for these insightful comments. Through the modification, we have made a more concise language expression and shortened the word count of the full text.

**Detailed comments:**

3. The authors should clarify whether "consecutive" patients were included in the study (or not).

**Response:** As **“**consecutive” patients were included in the study, so we clarified it in the article in line 31 and 119.

4. Please describe the inclusion and exclusion criteria more in detail. I suggest using a dedicated table for this purpose.

**Response:** Thank you for the useful comment. Now we use a dedicated table to describe the inclusion and exclusion criteria more in detail. See line 124 (Table 1) in the article.

5. The retrospective design of the study should be acknowledged among the limitations.

**Response:** The retrospective design of the study has been acknowledged among the limitations, see line 371-377 for details in the Discussion.

6. Single-center design. The present study has a single-center design and is limited to Chinese patients. This is a limitation that should be acknowledged. Interestingly, the characteristics and prevalence of cardiovascular risk factors in this Asian population are similar to those of other large contemporary trials (BMJ. 2021 Jun 16;373:n1332. doi: 10.1136/bmj.n1332 [PMID: 34135011]) and real-world registries (Angiology. 2018 Oct;69(9):795-802. doi: 10.1177/0003319718760917 [PMID: 29544348]) including other ethnicities. This is a relevant finding and potentially supports the generalizability of the results. I recommend discussing this point separately by adding the above-mentioned references to support this concept.

**Response:** Thank you for these insightful comments. We recognized that this is a single-center design and is limited to Chinese patients, further studies involving different populations and more centers are needed to support our findings. But the characteristics and prevalence of cerebrovascular and cardiovascular risk factors in the Asian population are similar to those of other large contemporary trials and real-world registries that also include other ethnicities. We discussed this point separately by adding the above-mentioned references to support this concept. See line 371-377 for details in the Discussion.

7. Mortality was assessed at 3-month follow-up. Therefore, long-term effects of serum TBA cannot be determined in this study. Please discuss.

**Response:** Thank you for the useful comment. Long-term effects of serum TBA cannot be determined in this study as long-term follow-up has not yet been completed. Further studies will be conducted to reveal the correlation between TBA and long-term prognosis of AIS patients. We explained this in the limitations, see 382-383 in the Discussion.

8. The authors should provide more detailed description on how the multivariable model was prepared.

**Response:** Thank you for your suggestion and now we provide more detailed description on how the multivariable model was prepared in Statistical analysis, see line 194-199.

9. The authors should better discuss the practical implications of their study.

**Response:** Thank you for the insightful comment. Now we discussed the practical implications of the study in Discussion and Conclusion. Please see line 354-361 for details in the Discussion and line 390-395 for details in the Conclusion.

10. The graphical presentation should be improved, for example, by adding an illustration summarizing the main findings of the study.

**Response:** Thank you for your suggestion and we now have a brief summary of the main findings of the study behind the Table.

**Reviewer #2:**

1. It should be stated whether the patients are taking lipid-lowering therapy or any medication that may cause cholestatic hepatitis.

**Response:** Thank you for the insightful comment. We admit that we did not record whether the patients were undergoing lipid-lowering therapy at the time of admission, and we are unable to obtain this information now because our data came from the medical record system. But all patients included in the study were not receiving any medications that may cause cholestatic hepatitis and ursodeoxycholic acid and other medications. See line 136-137 for details in Study population. We will record in more detail the lipid-lowering drugs taken by patients and whether the patients are taking lipid-lowering therapy or any medication that may cause cholestatic hepatitis in future researches.

2. Posthoc analysis should be performed for parameters found to be significant by ANOVA and Kruskal Wallis tests.

**Response:** Thank you for the useful suggestion. There were significant differences in AF history and WBC count at admission among the four groups. Posthoc analysis showed that that there was no significant difference in the AF rate between the Q1 and Q2 groups, while there was a significant difference between the other groups. The WBC count difference between the Q1 group and Q4 group was statistically significant (*P* < 0.05) and the WBC count in Q4 group was lower than in Q1 group (6.3 (5.1-7.9) vs 6.8 (5.6-8.5) ×10^9^/L). (Line 212-217)

3. Cox regression analysis should be preferred to determine parameters associated with mortality.

**Response:** Thank you for your suggestion. COX regression is the most suitable method for parameters related to mortality. However, we only reviewed whether the patient died within 3 months after the occurrence of AIS. We did not record when the patient died, so Cox regression analysis was not possible.

4. The strength and limitations section should be written in detail under a subtitle.

**Response:** Thank you for the useful comment. We have now detailed the strength and limitations section under the subtitle. Please see line 362-385 for details in the article.

**Reviewer #3:**

**General comments:**

A general comment is that manuscript should be review again for the journal rules. A general comment is that grammatical mispelling improvement is necessary throughout the manuscript by the journal' rules.

**Response:** Thank you for these constructive comments. The manuscript presentation has been improved (including the abstract) by a native speaker (AJE website) for typos and grammatical errors.

**Specific comments:**

1. Abstract: Please write full abbreviations in the abstract. Please write the method in more detail in the abstract.

**Response:** Thank you for your useful suggestion. The full abbreviations and the more detailed methods were written in the abstract now. Please see line 33-35 for details.

2. Methods: Please added ethics approved date and number.

**Response:** The clearance No for Ethical approval is No. 2020-77-1. Please see Line 145 in the article.

3. Discussion: Please removed line 290-298.

**Response:** Thank you for your suggestion and we removed line 290-298.

4. Conclusion: This section is not enough. Authors must be impressed future clinical implications.

**Response:** Thank you for the insightful comment. Now we discussed the practical future clinical implications of the study in Discussion and Conclusion. Please see line 354-361 for details in the Discussion and line 390-395 for details in the Conclusion.
